# Inter-reader variability of SPECT MPI readings in low- and middle-income countries: Results from the IAEA-MPI Audit Project (I-MAP)

**DOI:** 10.1007/s12350-018-1407-4

**Published:** 2018-08-30

**Authors:** Maurizio Dondi, Carlo Rodella, Raffaele Giubbini, Luca Camoni, Ganesan Karthikeyan, Joao V. Vitola, Andrew J. Einstein, Bertjan J. Arends, Olga Morozova, Thomas N. Pascual, Diana Paez, the I-MAP investigators, M. Beretta, N. Better, S. Bouyoucef, L. O. Cabrera Rodríguez, G. Chalal, C. Cittanti, C. Cruz, A. Cuocolo, N. Girotto, N. T. Huong, S. S. Iqbal, A. Klaipetch, C. Marcassa, E. Milan, F. Mut Bastos, Q. Naïli, D. Nanayakkara, J. Obaldo, T. F. Ouattara, Faso Burkina, K. M. Padrón García, A. Peix, Y. Peña, N. Y. Poyraz, M. Prpic, A. Rochela, D. F. Ruiz Castañeda, R. Sciagra, S. Scotti, E. Sereegotov, S. Sestini, D. Sobic Saranovic, J. Spuler, T. Thientunyakit, W. Vangu, J. Vitola, G. Vuleta

**Affiliations:** 1 Nuclear Medicine Department, University of Brescia, Brescia, Italy; 2 Department of Medical Physics, Spedali Civili di Brescia, Brescia, Italy; 3 Department of Cardiology, All India Institute of Medical Sciences, New Delhi, India; 4 Quanta Diagnóstico & Terapia, Curitiba, Brazil; 5 Department of Medicine, Division of Cardiology, and Department of Radiology, Columbia University Irving Medical Center, New York, USA; 6 Department of Medical Physics, Catharina Hospital, Eindhoven, The Netherlands; 7 Nuclear Medicine and Diagnostic Imaging Section, Division of Human Health, International Atomic Energy Agency, Vienna, Austria

**Keywords:** CAD, Myocardial ischemia and infarction, Gated SPECT, Image interpretation

## Abstract

**Background:**

Consistency of results between different readers is an important issue in medical imaging, as it affects portability of results between institutions and may affect patient care. The International Atomic Energy Agency (IAEA) in pursuing its mission of fostering peaceful applications of nuclear technologies has supported several training activities in the field of nuclear cardiology (NC) and SPECT myocardial perfusion imaging (MPI) in particular. The aim of this study was to verify the outcome of those activities through an international clinical audit on MPI where participants were requested to report on studies distributed from a core lab.

**Methods:**

The study was run in two phases: in phase 1, SPECT MPI studies were distributed as raw data and full processing was requested as per local practice. In phase 2, images from studies pre-processed at the core lab were distributed. Data to be reported included summed stress score (SSS); summed rest score (SRS); summed difference score (SDS); left ventricular (LV) ejection fraction (EF) and end- diastolic volume (EDV). Qualitative appraisals included the assessment of perfusion and presence of ischemia, scar or mixed patterns, presence of transient ischemic dilation (TID), and risk for cardiac events (CE). Twenty-four previous trainees from low- and middle-income countries participated (core participants group) and their results were assessed for inter-observer variability in each of the two phases, and for changes between phases. The same evaluations were performed for a group of eleven international experts (experts group). Results were also compared between the groups.

**Results:**

Expert readers showed an excellent level of agreement for all parameters in both phase 1 and 2. For core participants, the concordance of all parameters in phase 1 was rated as good to excellent. Two parameters which were re-evaluated in phase 2, namely SSS and SRS, showed an increased level of concordance, up to excellent in both cases. Reporting of categorical variables by expert readers remained almost unchanged between the two phases, while core participants showed an increase in phase 2. Finally, pooled LVEF values did not show a significant difference between core participants and experts. However, significant differences were found between LVEF values obtained using different software packages for cardiac analysis.

**Conclusions:**

In this study, inter-observer agreement was moderate-to-good for core group readers and good-to-excellent for expert readers. The quality of reporting is affected by the quality of processing. These results confirm the important role of the IAEA training activities in improving imaging in low- and middle-income countries.

**Electronic supplementary material:**

The online version of this article (10.1007/s12350-018-1407-4) contains supplementary material, which is available to authorized users.

## Introduction

The International Atomic Energy Agency (IAEA) is an independent, intergovernmental science and technology-based organization which is part of the United Nations family of organizations.^1^ The IAEA works with its 170 Member States (MS) and multiple partners worldwide to promote the safe, secure and peaceful use of nuclear technologies. The IAEA supports nuclear medicine through activities of the Nuclear Medicine and Diagnostic Imaging Section (NMDI) within a quality assurance framework.^2^,^3^ The nuclear medicine programme contributes to achieving the sustainable development goals (SDGs) set by the United Nations, one of which is “by 2030, reduce by one third premature mortality from non-communicable diseases through prevention and treatment and promote mental health and well-being”.^4^

Considering the burden of cardiovascular diseases (CVD) as a major threat to public health worldwide,^5^,^6^ and the important role of nuclear techniques such as myocardial perfusion imaging (MPI) in the management of patients with ischemic heart disease (IHD),^7^-^9^ the NMDI Section adopted a strategic decision of strengthening capacity building in nuclear cardiology (NC), providing training through national and regional projects,^10^ supported by the Technical Cooperation Programme (TCP), which is the IAEA’s main mechanism for transferring nuclear technology to Low- and Middle-Income Countries (LMICs).^11^ Educational activities in NC include several Regional Training Courses (RTC) carried out over the past ten years.

This paper reports the results of an audit of NC practices (the I-MAP study), initiated in 2015 to assess whether and how training provided through RTCs impacted the quality of clinical practice. The primary goal was to assess homogeneity (i.e. intra- and inter-observer variability) within a group of core participants from LMICs. As secondary goals the study aims at a) evaluating the impact of IAEA activities in NC; b) comparing the readings of MPI studies in limited resource centres with those of international experts; c) evaluating the quality of reporting and d) assessing the impact of the reconstruction of MPI studies on the quality of reporting.

## Methods

**Figure 1:**
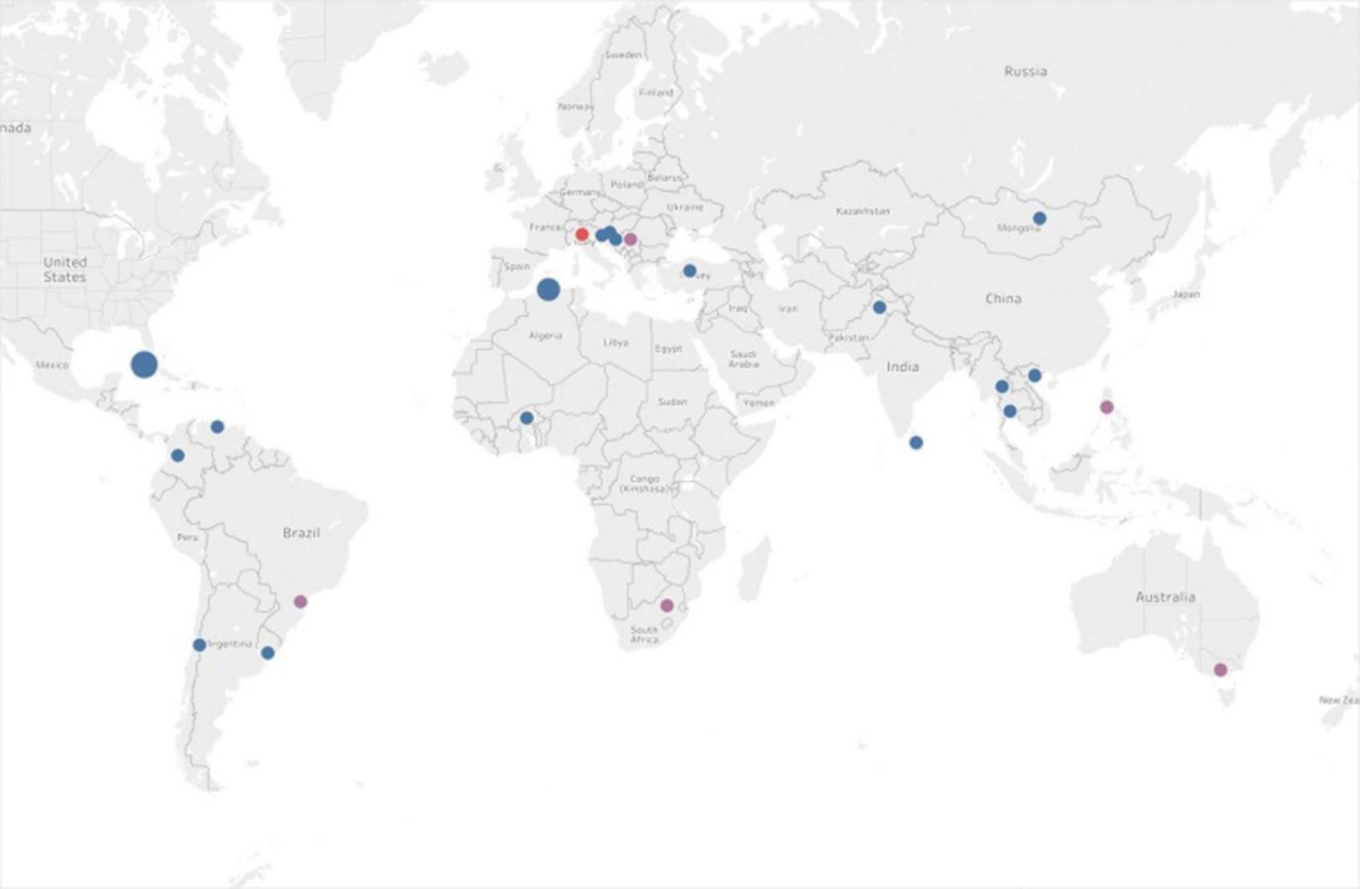
Worldwide distribution of both participants and experts. Red dot identifies the Core Lab (Nuclear Medicine Dept, University of Brescia). The size of dots reflects more than one participant from the same country

Recorded contact data from all attendees to RTCs in NC was retrieved. In the preceding 10 years, 896 participants had attended a total of 41 RTCs. Their regional distribution is reported in Appendix (Table 5). To make sure that those trainees, prospective participants to this study, were still actively involved in NC, that list was cross-checked with data from an international database managed by the IAEA.^12^ Of the 896 participants, 275 were identified as being currently active as nuclear cardiologists, and were approached for potential participation this study. Of these, 24/275 (8.7%), participated in the study. They formed the group referred to as “core participants.” Figure 1 reports their distribution around the world. The “core participants” group included physicians trained in nuclear medicine, with limited formal training in nuclear cardiology, in most cases acquired through short-term fellowships supported by the IAEA and/or trained “on the job.” Their yearly average volume of MPI studies was 880, with a minimum of 559 and a maximum of 1200.

The second group of “expert readers” consisted of eleven international experts identified by the Agency from a pool of its consultants and lecturers, and internationally recognized nuclear cardiologists. Overall, for the experts, the yearly volume of SPECT-MPI studies was on average double that of the core participants.

Both core participants and expert readers were requested to report anonymized case studies provided by a Core Lab, chosen on the basis of sound NC practice and significant record of research. The core lab identified 15 studies which, after anonymization, were uploaded onto a cloud-based collaborative platform (SharePoint™) and then downloaded from both core participants and experts.

All studies were carried out with the two-day protocol, using Tc99m labelled perfusion agents, and patients were imaged only in supine position. To provide readable studies for centres with limited technical resources, the core lab was asked to send studies processed with neither resolution recovery, nor scatter or attenuation correction, nor studies acquired with CZT cameras. Clinical data, including patients’ history, rest and stress ECG recordings and symptoms during stress were made available to participants. Relevant demographic and clinical data are summarized in Table 1.

**Table 1:** Patients’ data and clinical status

	Males	Females
#	9	6
Age (mean)	71.1	64.5
BMI (mean)	26.4	29.8
Mean HR at rest (bpm)	64.3	74.8
Mean systolic BP	141.1	150.8
Mean diastolic BP	87.8	91.7
Previous MI (yes/no)	5/2 + 2 CABG	3/3
Stressor (Ex/Pharm)	4/5	1/5
Symptoms at peak stress (yes/no)	5/4	4/2

*BMI*, Body Mass Index; *HR*, heart rate; *BP*, blood pressure; *MI*, myocardial infarction; *Ex*, exercise stress test; *Pharm*, pharmacologic stress test; *CABG*, Coronary Artery by-pass graft

**Figure 2:**
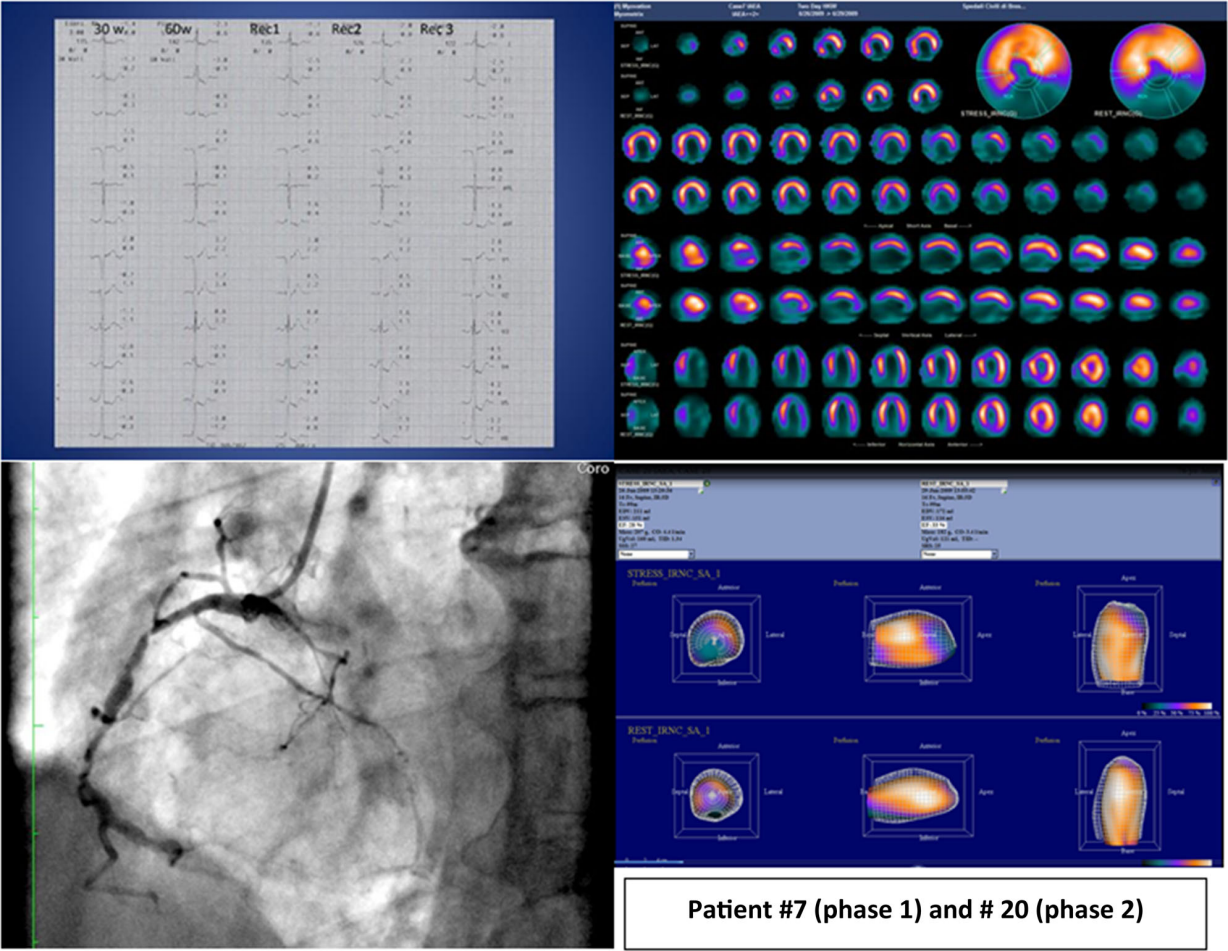
Example of a case study as distributed in phase 2: Male; 76 y-o; Family history of CAD; Hypertensive; Inferior AMI in 1970; relapse 1 year later; 1991 Coro: 75% stenosis RCA; distal occlusion RCA; 75% stenosis distal LAD; occlusion D2; patent LCX; OMT until 2009; Referred for MPI in 2009; Bicycle exercise; max workload 60 W for 3’; typical chest pain; ECG: inferior-lateral ST downslope and runs of NSVT. *CAD*, Coronary artery disease; *AMI*, acute myocardial infarction; *RCA*, right coronary artery; *LCX*, left circumflex; *OMT*, optimized medical therapy; *MPI*, myocardial perfusion imaging; *NSVT*, non-sustained ventricular tachycardia

We designed I-MAP to be run in two phases. In Phase 1, all 15 patient studies were provided as raw data. Both groups were requested to process them according to their own routine practice. For Phase 2, the same 15 cases were re-submitted in a different order, but pre-processed at the core lab using Myovation v3 software (GE Health Care; Haifa, Israel) with an iterative reconstruction ordered subset expectation maximization algorithm (2 iterations, 10 subsets) and motion correction. The “cool” GE colour scale was applied for tomographic slices representation. Both groups of participants were unaware that they were re-reading the same studies. This second phase was aimed at assessing whether reconstruction could have any impact on the overall quality of the study and consistency of interpretation. An example of a pre-processed patient study, as distributed in phase 2, is illustrated in Figure 2.

**Figure 3:**
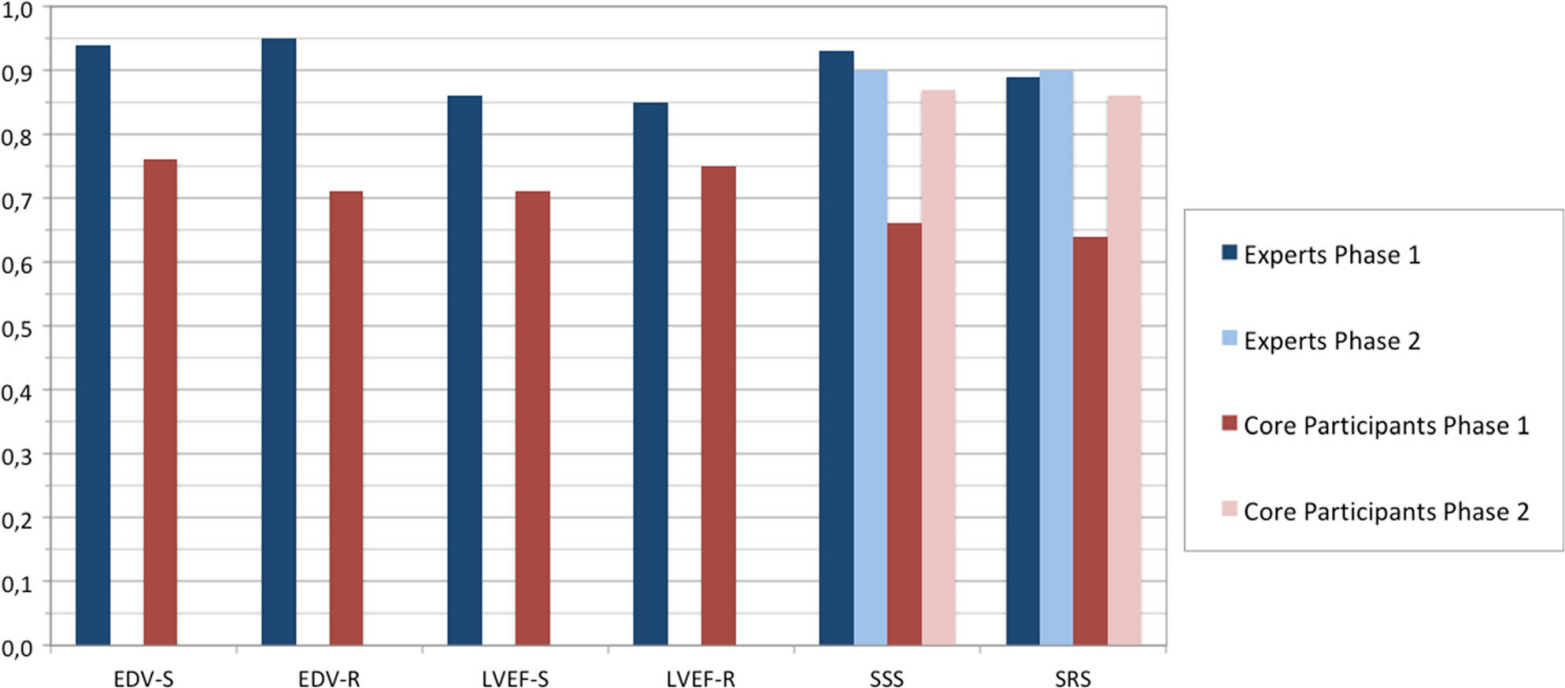
ICC values for continuous variables (EDV, LVEF, SSS, SRS). Calculation of EDV and LVEF was not requested for phase 2. *EDV-S/R*, end-diastolic volume post-stress/at rest; *LVEF-S/R*, left Ventricle Ejection Fraction post stress/at rest; *SSS*, summed stress score; *SRS*, summed rest score

We used standardized forms for data collection which were forwarded to the core lab for statistical analysis. After on-site processing for phase 1, and based on images provided by the core lab for phase 2, readers were requested to score tracer uptake in polar maps using a 17-segment model (Figure 3A). An important distinction is that while in phase 1 readers could accept any score given by the cardiac software, in phase 2 they had to digit their own interpretation. The severity of perfusion defects in each of the 17 myocardial segments, as defined by the American Heart Association^13^ is scored on a 0-4 scale.

Data to be reported included quantitative perfusion metrics such as Summed Stress Score (SSS); Summed Rest Score (SRS); and Summed difference Score (SDS). SDS results were pooled to generate three categories: (a) SDS ≤ 3; (b) 4 ≤ SDS ≤ 7 and (c) SDS ≥ 8.^14^.

For left ventricular function, quantitative data were reported on Left Ventricular Ejection Fraction (LVEF) and End Diastolic Volume (EDV), while regional wall motion was reported based on visual assessment. Other qualitative, or visual, appraisals included the assessment of perfusion, classified as normal or abnormal. In this latter case, readers had to report presence of ischemia, scar or mixed patterns. Another parameter visually analysed was presence or absence of Transient Ischemic Dilation (TID). Both groups were also requested to provide an overall judgment about patients being at high risk or not (PHR).

Furthermore, we aimed at assessing the relationship between the overall judgment of the status of perfusion, either normal or abnormal, and uptake scores (SSS; SRS) as the sum of scores assigned to each single segment. To this purpose and to avoid the possibility that high SSS values could just be the result of the sum of mild defects scattered throughout the myocardial wall, not representing significant perfusion defects, we defined “hypoperfusion cluster” as the presence of a real perfusion defect, when two adjacent segments scored ≥2. Then, we assessed the relationship between SSS values and the number of hypoperfusion clusters identified in the polar maps.

To evaluate the inter-reader concordance of hypoperfusion assessments, SDS values were stratified into three categories, a) SDS ≤3; b) 4 ≤ SDS ≤ 7 and c) SDS ≥ 8.^14^ For each study, each group of readers (both experts and core participants), and for both phase 1 and 2, the rate of responses for each of the three different SDS categories, was evaluated. These three categories have been called “SDS strat.”

For phase 1 we also tested the consistency of quantitative data, such as LVEF and EDV, since they were calculated using different software. This evaluation was run only for phase 1, since in phase 2 participants were provided pre-processed studies. Variables LVEF post stress and LVEF rest were analyzed using univariate analysis of variance (ANOVA).

Finally, we tested the repeatability of LVEF values when different processing software was used. To avoid the increased risk of Type I errors because of the multiple simultaneous hypotheses being tested, we adjusted *P* values using the Bonferroni method.^15^

### Statistical Analysis

For statistical analysis, data were collected on Excel spread sheets and analysed using the Statistical Package for Social Sciences (SPSS; IBM® SPSS® Statistics Release 24); For hypothesis testing, Student’s t-test, analysis of covariance (ANCOVA), ANOVA, and Chi-square test for proportions were used as appropriate, the latter for assessing difference in response rates between groups and phases. Intra-rater and inter-rater agreement were assessed:

- by means of the intra-class correlation coefficient (ICC), for continuous measurements (EDV, LVEF, SSS, SRS, SDS). ICC is a measure of agreement that combines information on both the correlation and the systematic differences between readings^16^,^17^; using ICC, the level of agreement is classified into four categories
- by means of the Fleiss’ kappa, for categorical variables (Function, Perfusion, TID, SDS strat, patient high risk). Using Fleiss’ kappa (*κ*) scores, the level of agreement is classified into seven categories.^18^-^20^

Values for either SSS and SDS reported from the two groups in phase 1, when MPI studies were supplied as raw data and each participant had to completely process and assess using their own software, were compared with those reported from phase 2, where studies were supplied pre-processed at the core lab and participants had to visually score segmental perfusion.

## Results

For continuous variables (EDV; LVEF; SSS; SRS) ICC values and the corresponding concordance category are reported in Figure 3 and in Table 2.

**Table 2:** ICC results (values and category of agreement)

Parameter	Experts				Participants			
	Phase 1		Phase 2		Phase 1		Phase 2	
EDV post stress	0.94	(excellent)	–	–	0.76	(excellent)	–	–
EDV rest	0.95	(excellent)	–	–	0.71	(good)	–	–
LVEF post stress	0.86	(excellent)	–	–	0.71	(good)	–	–
LVEF rest	0.85	(excellent)	–	–	0.75	(excellent)	–	–
SSS	0.93	(excellent)	0.90	(excellent)	0.66	(good)	0.87	(excellent)
SRS	0.89	(excellent)	0.90	(excellent)	0.64	(good)	0.86	(excellent)

Metrics for EDV and LVEF are assessed only for phase 1, as in phase 2 these data were already calculated at the Core lab. Expert readers showed an excellent level of agreement for all parameters in both phase 1 and 2, spanning from 0.85 for LVEF at rest to 0.94 for EDV post-stress. In phase 1, concordance levels for core participants were rated as good for all parameters (from 0.64 to 0.71), except for LVEF at rest and EDV post stress, which were rated as excellent (0.75 and 0.76, respectively). Interestingly, both parameters which were re-evaluated in phase 2, i.e. SSS and SRS, showed an increased level of concordance, up to 0.87 and 0.86 (excellent).

**Figure 4:**
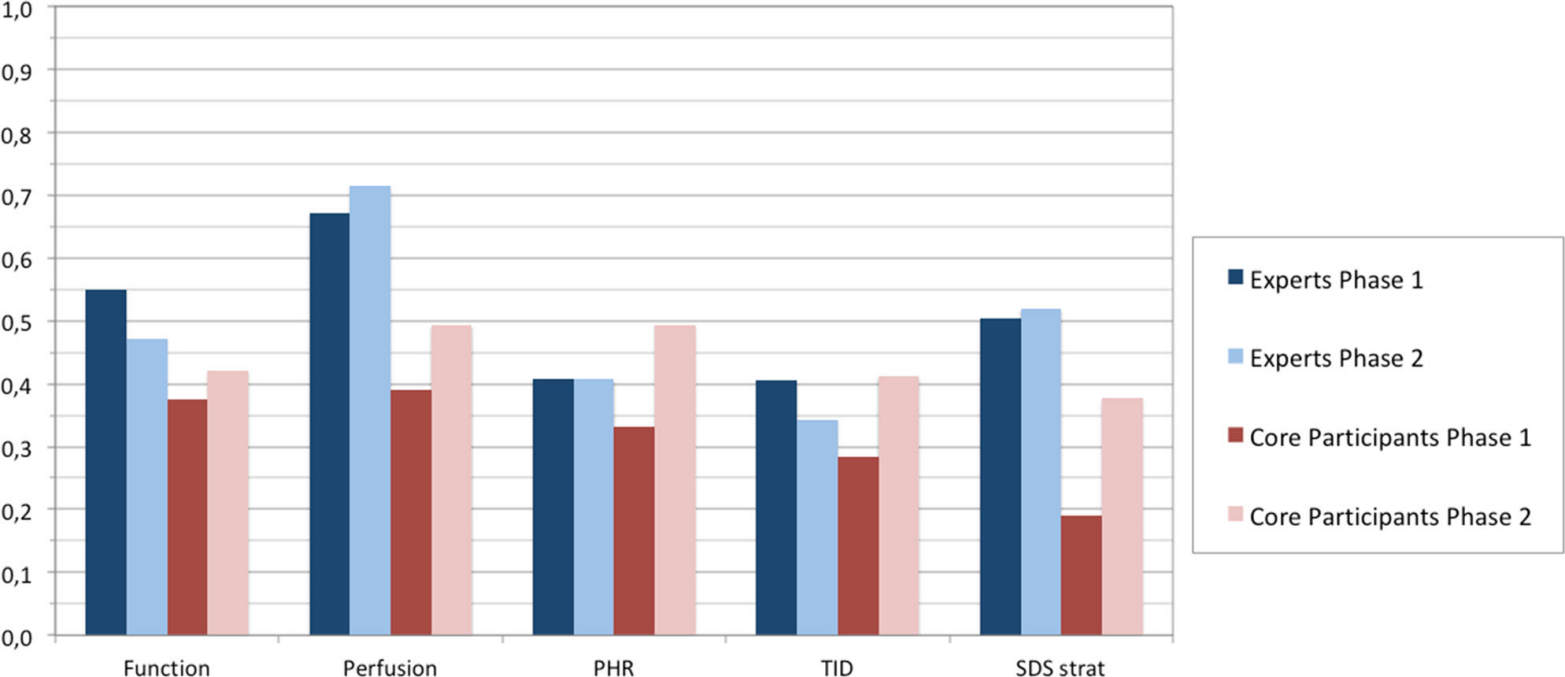
Calculated Fleiss’ kappa values for categorical variables. *TID*, Transient Ischemic Dilation; *SDS*, Summed Differential Score stratified; *PHR*, patient high risk)

Fleiss’ kappa values for categorical variables are summarized in Figure 4 and Table 3, along with the significance of concordance. In this case, reports from phase 1 and 2 are compared for all variables. For those variables, categories of agreement for expert readers between the two phases remained almost unchanged, with the exception of TID, while core participants showed an increase for all variables.

**Table 3:** Fleiss’ kappa results (values and category of agreement)

Parameter	Experts				Participants			
	Phase 1		Phase 2		Phase 1		Phase 2	
Function	0.55	(moderate)	0.47	(moderate)	0.38	(fair)	0.42	(moderate)
Perfusion	0.67	(substantial)	0.71	(substantial)	0.39	(fair)	0.49	(moderate)
PHR	0.41	(moderate)	0.41	(moderate)	0.33	(fair)	0.49	(moderate)
TID	0.41	(moderate)	0.34	(fair)	0.28	(fair)	0.41	(moderate)
SDS strat	0.50	(moderate)	0.52	(moderate)	0.19	(slight)	0.38	(fair)

*PHR*, patient high risk; *TID*, transient ischemic dilation; *SDS strat*, stratification of perfusion defects

**Figure 5:**
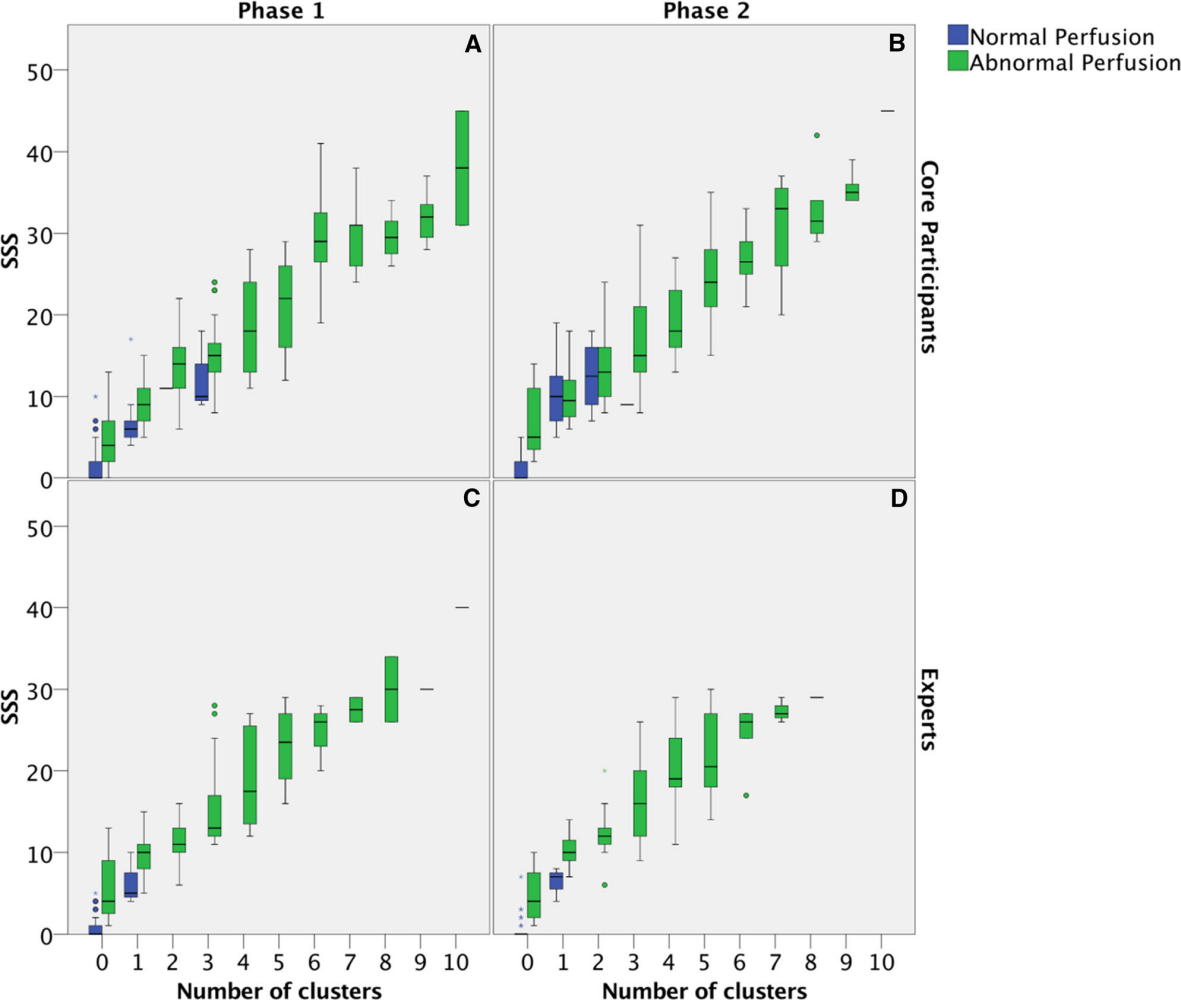
SSS value as function of the number of hypoperfusion clusters. Green boxes represent patients whose perfusion has been judged as abnormal. Blue boxes represent normal perfusion judgments. (**A**) Experts phase 1, (**B**) Experts phase 2, (**C**) Core participants phase 1, (**D**) Core participants phase 2. The figure shows box-and-whiskers plot, showing the median, quartiles, and outlier and extreme values for a scale variable. The interquartile range (IQR) is the difference between the 75th and 25th percentiles and corresponds to the length of the box. Circles outside the boxes represent OUTLIERS. Outliers are values between 1.5 IQR’s and 3 IQR’s from the end of a box. Stars represent EXTREME whose values are more than 3 IQR’s from the end of a box

Relationship between SSS values as reported from both experts and core participants and the number of “hypoperfusion clusters”, as derived from polar maps, is summarized in Figure 5. In more detail, Figures 5A and B represent results from experts in phases 1 and phase 2, respectively; while in Figures 5C and D the same analysis is reported for Core participants.

If we consider SSS mean values as a function of cluster number and then we determine a linear interpolation between the experimental data, we observe a tendency towards statistical significance (F=3.64 and p=0.057) for curve slopes only between phase 1 and phase 2 for core participants (Table 4).

**Table 4:** Linear interpolation slopes for average SSS values vs cluster numbers

		ANCOVA		
		Linear regression slope coefficient comparison		
Comparison	Items	Phase 1	Phase 2	Test results
Phase 1 vs Phase 2	Experts	Slope = 3.940	Slope = 4.068	*F* = 0.90
		*R*^2^ = 0.831	*R*^2^ = 0.835	*P* = 0.533
		*s*_slope_ = 0.141	*s*_slope_ = 0.149	
	Core participants	Slope = 3.756	Slope = 4.019	*F* = 3.64
		*R*^2^ = 0.836	*R*^2^ = 0.830	*P* = 0.057
		*s*_slope_ = 0.087	*s*_slope_ = 0.110	
Comparison	Items	Experts	Core participants	Test results
Experts vs core participants	Phase 1	Slope = 3.940	Slope = 3.756	*F* = 1.22
		*R*^2^ = 0.831	*R*^2^ = 0.836	*P* = 0.270
		*s*_slope_ = 0.141	*s*_slope_ = 0.087	
	Phase 2	Slope = 4.068	Slope = 4.019	*F* = 0.06
		*R*^2^ = 0.835	*R*^2^ = 0.830	*P* = 0.801
		*s*_slope_ = 0.149	*s*_slope_ = 0.110	

As already described, based on SDS values, patients have been stratified (SDS-strat) as “low risk” (SDS ≤3); “intermediate risk” (4 ≤ SDS ≤ 7) and “high risk” (SDS ≥ 8), according to their SDS value. Analysing differences in risk stratification as described by SDS values between phases, we found that there is a significant difference for SDS strat between phases 1 and 2, for 3 studies out of 15 in the core participants group, and in 2 of 15 in experts.

As already described, participants were encouraged to analyze and report submitted studies according to their daily routine, including use of their cardiac software. Well aware of the possible impact on calculated values such as LVEF and EDV, information was also collected on type of cardiac software utilized. For both Core participants and Experts, the distribution of the different cardiac software available on the market is reported in Appendix (Table 6).

Evaluations on LVEF values included the following factors: Group (2 levels: Experts, Core Participants); cardiac software (5 levels: 4DMCardio; CedarsSinai; EmoryCardiacToolBox; InterView; Other); Case Study number (15 levels: patient studies 1-15). Changes in variables were assessed as a function of factors and interaction between factors themselves. Results are shown in Table 7 of the Appendix. There were significant differences in the LVEF values calculated both post-stress and at rest and for values calculated from the different types of software. The Bonferroni post-hoc analysis of multiple comparisons shows that one of the software packages (EmoryCardiacToolBox) systematically produces an LVEF value significantly lower than 4DMCardio, CedarsSinai, and Other software (range of differences: − 8.2% to − 10.8%); while no significant differences are found with the InterView software (see Table 8 in Appendix for details).

**Figure 6:**
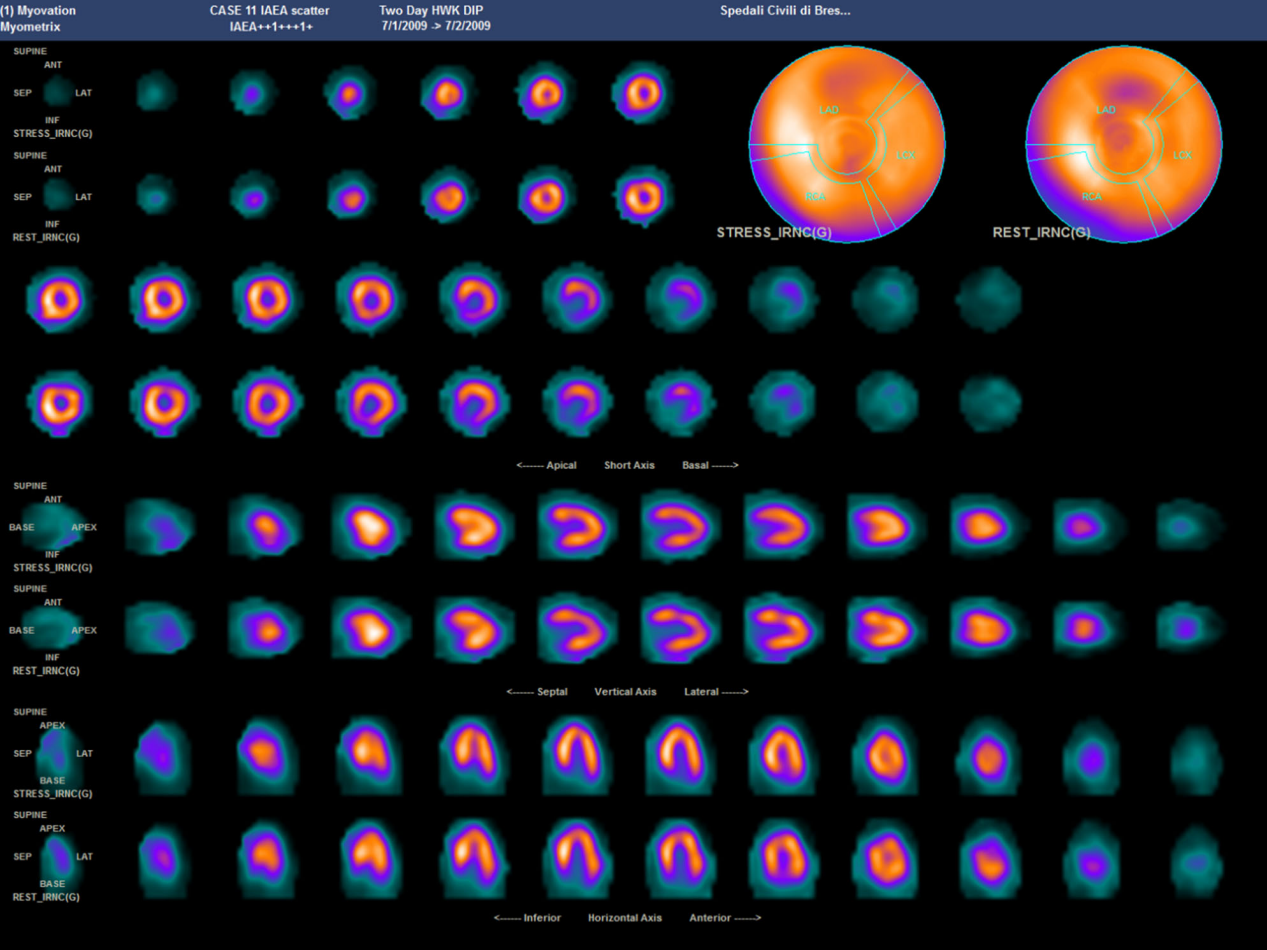
Case #11 of phase 1: Female, 71 y-o; Type 2 diabetes on medication with metformin; Hypertension treated by vasodilators (Enalapril); 5 yrs before MPI cardioversion for atrial fibrillation. On chronic therapy with warfarin and propafenone; H 162 cm W 79 kg; Referred for chest pain not related to efforts; Dipyridamol stress test (0.84 mg/kg/5 minutes); Rest BP 170/95, 67 BPM; At the end of Dipyridamol infusion BP = 150/100, 95 BPM

Overall, LVEF post-stress values are not significantly different between core participants and experts (Table 9 in Appendix). Average SD levels for the readings of core participants were about twice as high as the average SD levels for the experts group (10.4% vs 5.8%), a finding which was also expressed in the higher ICC for the latter group (Figure 3). Case 11 that caused relatively larger SD values in both core participants and experts readers groups (18.5 and 19.4, respectively) is represented in Figure 6.

## Discussion

Often, in medical imaging, interpretation of results is subjective^21^-^30^ and can be influenced by technical considerations. Quality plays a pivotal role when analysing and reporting an imaging study. Several factors can affect the results of the analysis and the value of the studies. This is true for all modalities and in the case of SPECT MPI,^31^-^35^ which is the subject of this study, it is crucial to ensure that the acquisition and reconstruction parameters are consistent and optimized, thus allowing accurate and reproducible results.

Several factors, in different phases of the procedure, might influence the final results of MPI studies and require scrutiny. They include, but are not limited to, pre-examination checks, such as appropriateness of reference, QA/QC of equipment and radiopharmaceutical preparation, to steps to be taken during examination, such as QA/QC of acquisition parameters and of processing and reporting. We geared the I-MAP study towards assessing the quality of processing and reporting.

We examined the reliability of SPECT MPI studies using inter-observer variability within two groups of participants: one made of practitioners from LMICs, which are indeed the target of IAEA’s educational activities, and a second group of expert readers. The first group of “core participants” was composed of nuclear cardiology professionals who attended training events managed by the IAEA, many of them working in settings where financial resources might be limited, therefore with limited experience and limited resources for improving their expertise. As regards the study, it was run in two phases and in both of them participants had to report the same group of 15 cases, with the important difference that in phase 1 all participants were provided raw data and were requested to process them according to their routine practice and then report. In phase 2, all participants were given, in different order, the same 15 cases pre-reconstructed and were requested to provide their segmental uptake score, visually assessed, as well as other qualitative interpretations. Both groups were unaware that in phase 2 they were re-evaluating the same studies.

For quantitative data such as EDVs and LVEFs, an excellent level of concordance was found within both groups for both phase 1 and 2 (Table 2; Figure 3). Concordance was also excellent within the experts group for SSS and SRS values in both phases.

It’s very Interesting that, for the latter two parameters (SSS and SRS), core readers showed an excellent intra-group agreement in phase 2 when they had to provide their own evaluation on pre-processed images (0.87 and 0.86; for SSS and SRS respectively), while in phase 1, when they had to process the studies and scores were automatically calculated by their software, concordance was only good, being 0.66 and 0.64; for SSS and SRS respectively).

It should be remembered that while in phase 1 readers could accept segmental scores from their own software, or override if needed, in phase 2 scores had to be visually assessed and manually entered into the forms, therefore reflecting a qualitative rather than a semi-quantitative evaluation. Therefore, we relate this improvement to the central role of processing: when less experienced readers are presented with well processed studies and are forced to score perfusion status, their readings are as good as experts’ readings. This finding confirms that processing remains a crucial step for the overall SPECT MPI evaluation and that experience and training plays a major role for good quality processing. Furthermore, this finding tells us that, besides physicians who actually are those who read studies, IAEA training events should also involve technologists who often perform the processing.

Further confirmation of the importance of processing is found when we compare performances between the two groups for risk stratification. In this case, when we analysed differences between the experts panel and the core participants group, we have found that in phase 1 a significant difference could be seen in 2/15 cases, while no difference could be seen between the two groups for phase 2, when the core lab distributed pre-processed studies.

Fleiss’ kappa value is a rather stringent index, very sensitive to even small deviations between readers which may cause an important worsening of calculated values. In this study, it showed that experts, as expected, had a greater concordance in interpretation, in both phases of the study, while for core participants concordance improved significantly between phase 1 and 2. This finding holds true for both the analysis of continuous variables and for SSS and SRS indexes. Once more, this finding supports the notion that interpretation in itself is not the issue, but what is going to be interpreted is. When study processing is not properly carried out, then interpretation suffers.

A tendency of core participants to give an overall evaluation of “normal perfusion” even in presence of significant SSS values and hypoperfusion clusters was observed (Figure 5).

The greater variability in interpreting on-site processed images, as requested in phase 1, might well be affected by poor alignment of slices because of bad selection of left ventricular axes, valve planes and apex. So, while experts were able to minimize the impact of processing on the quality of images, this was not the case for core participants, who indeed markedly improved their performance when they were given studies which had been pre-processed at the core lab. Pre-processing included motion correction, careful slice realignment between stress and rest acquisitions, correct choice of slice thickness to avoid artefacts due to partial volume effect, and correct colour scale levelling in presence of extracardiac hot-spots such as sub-diaphragmatic activity.

Finally, we found, as reported by other groups^36^,^37^ that important parameters such as LVEF, calculated through gated SPECT, may differ significantly when different processing software packages are used, as shown in Table 8. One software deviates substantially and significantly from almost all the other software packages, with a systematic bias in LVEF of − 8.3% down to − 10.8% which could be clinically significant when LVEF is used in clinical decision making, such as in longitudinal studies of cardio-oncological patients.

The univariate analysis of variance for LVEF post-stress and LVEF at rest was run considering the different factors involved and their interactions. Results of that analysis reported in Table 7 also show significant differences for LVEF values calculated both post-stress and at rest, and for values calculated from the different types of software.

Overall, LVEF values are not significantly different between the two groups, core participants and experts, as shown in Table 9. A relatively wide SD shown for case #11 could be attributed to factors such as patient movement during acquisition (which could have been corrected for by readers), small heart with partial volume effect, hypertrophic left ventricular walls due to hypertension, and attenuation due to obesity (Figure 6).

## New Knowledge Gained

This study has shown that the quality of processing remains a crucial step for SPECT MPI and that experience helps overcome possible artefacts that may hamper the quality of reporting. As concerns the IAEA, this study shows that the outcomes of training events in NC are satisfactory, as the performance of NC professionals from LMICs does not differ significantly from expert readers in many circumstances, and particularly when good quality processing was applied to clinical studies. This latter consideration supports the concept that training courses should necessarily cover basic issues such as study processing. In addition, this study shows that LVEF values may differ significantly depending on the cardiac package employed and this should be kept in mind particularly when patients are studied in different institutions or when an institution adopts a different software package.

## Limitations of the Study

The small sample size of 24 participants from LMICs is a very low response rate for survey data, challenging the generalizability of findings. Furthermore, we don’t know to what extent “core participants” are representative of the reading pattern in LMICs. This is, however, unavoidable when dealing with centres from developing world because of difficult communication as well as technical problems affecting data transfers and report transmission, which may affect active participation.

One more important limit of the study design is the choice of not requiring participants to provide images along with reporting forms. This choice was made to minimize image transmission problems, but prevented full quality checks from being performed for the processed studies.

## Conclusions

The quality of reporting SPECT MPI could be rated as moderate-to-good for participants from emerging economies and good-to-excellent for expert readers. It is clearly affected by the quality of processing. Indeed, when readers with less experience are asked to report on studies pre-processed at an experienced core lab and by professionals well-trained to avoid sources of artefacts, inter-observer agreement between readers with less experience improves substantially. To our knowledge, this is the first study reporting these findings.

Significant differences were found between LVEF values obtained using different software packages for cardiac analysis. This should be kept in mind particularly when patients are studied in different institutions or when an institution adopts a different software.

This study calls for attention from scientific societies on the issue of the quality of study processing, suggesting the need for more stringent guidelines about this aspect of NC practice.

Finally, these results suggest that the outcomes of training events conducted by the IAEA in NC are satisfactory. However, in order to improve the quality of processing, future training courses should necessarily cover this issue, and should also involve technologists.

### Electronic supplementary material

Below is the link to the electronic supplementary material.

Supplementary material 1 (PPTX 275 kb)

## Appendix

See Tables 5, 6, 7, 8 and 9.

**Table 5:** Summary of training events managed by NMDI

Region	No. of training courses	No. of participants
Africa	8	155
Asia	10	208
Middle East	3	55
Eastern Europe	13	285
Latin America and Caribbean	7	193
Total	41	896

**Table 6:** Distribution of cardiac SW among both groups (core participants and experts)

Cardiac Software	Core participants (%)	Experts (%)
4DMCardio	28.4	9.4
CedarsSinai	43.0	90.6
EmoryCardiacToolBox	15.8	–
Interview	10.6	–
Other	2.3	–

**Table 7:** ANOVA univariate analysis of LVEF post stress and at rest for factors SW; group; case study number and their interactions

Source	LVEF post stress		LVEF rest	
	*F*	Sig.	*F*	Sig.
Software	12.041	**< 0.001**	11.772	**< 0.001**
Group	1.204	0.273	1.075	0.300
Case Study Number	28.384	**< 0.001**	23.657	**< 0.001**
Software × Group	0.048	0.826	0.500	0.480
Software × Case Study Number	1.293	0.096	0.719	0.922
Group × Case Study Number	0.316	0.992	0.334	0.989
Software × Group × Case Study Number	0.364	0.980	0.288	0.993

Highly significant *P* values are given in bold

**Table 8:** Bonferroni multiple comparisons post hoc test for the different cardiac SW utilized by study participants

Factor		LVEF post stress (%)			LVEF rest (%)		
Software (I)	Software (J)	Mean Difference (I–J)	SE	*P* value	Mean difference (I–J)	SE	*P* value
4DMCardio	CedarsSinai	− 0.017	1.059	1.000	0.350	0.972	1.000
	EmoryCardiacToolBox	− 8.266	1.560	**< 0**.**001**	− 8.244	1.555	**< 0**.**001**
	Interview	− 3.540	1.793	0.489	− 3.497	1.646	0.342
	Other	2.532	3.474	1.000	1.858	3.189	1.000
CedarsSinai	4DMCardio	0.017	1.059	1.000	− 0.350	0.972	1.000
	EmoryCardiacToolBox	− 8.248	1.399	**< 0.001**	− 8.594	1.420	**< 0.001**
	Interview	− 3.523	1.654	0.338	− 3.847	1.519	0.117
	Other	2.549	3.405	1.000	1.508	3.126	1.000
EmoryCardiacToolBox	4DMCardio	8.266	1.560	**< 0.001**	8.244	1.555	**< 0.001**
	CedarsSinai	8.248	1.399	**< 0**.**001**	8.594	1.420	**< 0**.**001**
	Interview	4.725	2.013	0.193	4.747	1.944	0.150
	Other	10.798	3.593	**< 0.001**	10.102	3.353	**< 0.001**
Interview	4DMCardio	3.540	1.793	0.489	3.497	1.646	0.342
	CedarsSinai	3.523	1.654	0.338	3.847	1.519	0.117
	EmoryCardiacToolBox	− 4.725	2.013	0.193	− 4.747	1.944	0.150
	Other	6.072	3.700	1.000	5.355	3.396	1.000
Other	4DMCardio	− 2.532	3.474	1.000	− 1.858	3.189	1.000
	CedarsSinai	− 2.549	3.405	1.000	− 1.508	3.126	1.000
	EmoryCardiacToolBox	− 10.798	3.593	**< 0.001**	− 10.102	3.353	**< 0.001**
	Interview	− 6.072	3.700	1.000	− 5.355	3.396	1.000

Highly significant *P* values are given in bold

**Table 9:** Comparison of results for LVEFs (phase 1 only) between experts and non-experts

Case study #	LVEF post-stress				
	Expert				*P* value
	No		Yes		
	Mean	SD	Mean	SD	
1	54.8	8.1	54.0	4.6	0.761
2	50.0	9.5	44.3	6.2	0.078
3	76.5	11.3	76.1	4	0.908
4	56.8	8.8	54.2	1.9	0.335
5	49.8	5.1	47.8	2.8	0.226
6	72.2	10.3	74.2	6.4	0.551
7	28.6	8.1	26.5	4.2	0.441
8	47.0	9.9	45.5	4.9	0.657
9	44.6	9.3	43.8	3.8	0.792
10	69.3	15.4	70.7	2.9	0.767
11	79.0	18.5	79.5	19.4	0.952
12	67.8	5.7	67.5	4.5	0.887
13	41.1	12.4	41.6	7.8	0.902
14	80.1	13.6	83.7	8.4	0.446
15	47.6	6.6	44.2	2.9	0.136

## Abbreviations

IAEA: International Atomic Energy Agency
MPI: Myocardial perfusion imaging
LVEF: Left ventricle ejection fraction
SSS: Summed stress score
SRS: Summed rest score
SDS: Summed difference score
EDV: End-diastolic volume
TID: Transient ischemic dilation
CE: Cardiac events
PHR: Patient high risk
