# Supplementary material for: Inter-reader variability of SPECT MPI readings in low- and middle-income countries: Results from the IAEA-MPI Audit Project (I-MAP)
Source: J Nucl Cardiol. 2018 Aug 30;27(2):465–78. doi: 10.1007/s12350-018-1407-4 (PMC7174263; doi:10.1007/s12350-018-1407-4)
Supplement: Supplementary file 1 — Supplementary material 1 (PPTX 275 kb) [file 12350_2018_1407_MOESM1_ESM.pptx]

## Slide 1
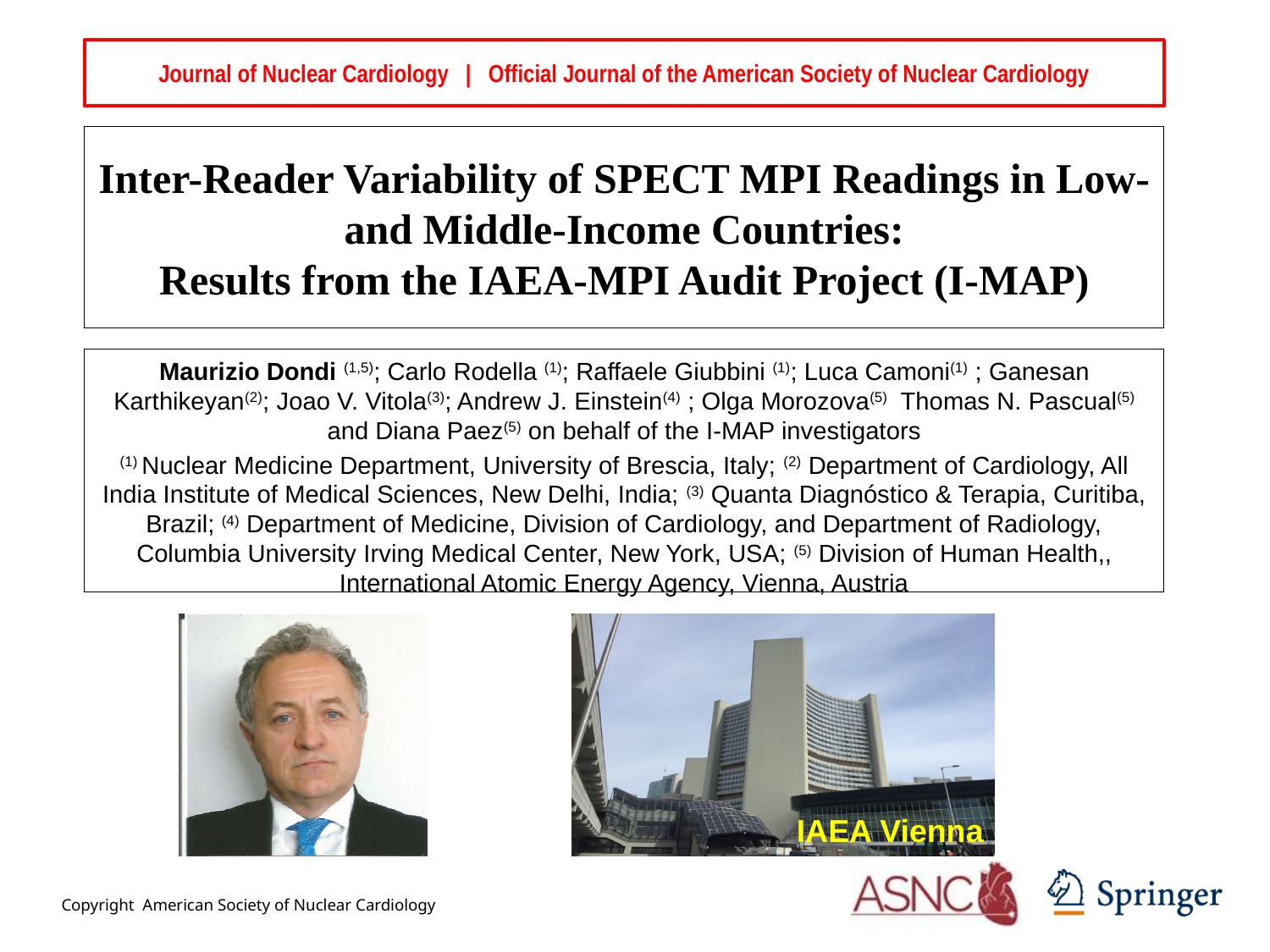

Journal of Nuclear Cardiology | Official Journal of the American Society of Nuclear Cardiology
# Inter-Reader Variability of SPECT MPI Readings in Low- and Middle-Income Countries: Results from the IAEA-MPI Audit Project (I-MAP)
Maurizio Dondi (1,5); Carlo Rodella (1); Raffaele Giubbini (1); Luca Camoni(1) ; Ganesan Karthikeyan(2); Joao V. Vitola(3); Andrew J. Einstein(4) ; Olga Morozova(5) Thomas N. Pascual(5) and Diana Paez(5) on behalf of the I-MAP investigators
(1) Nuclear Medicine Department, University of Brescia, Italy; (2) Department of Cardiology, All India Institute of Medical Sciences, New Delhi, India; (3) Quanta Diagnóstico & Terapia, Curitiba, Brazil; (4) Department of Medicine, Division of Cardiology, and Department of Radiology, Columbia University Irving Medical Center, New York, USA; (5) Division of Human Health,, International Atomic Energy Agency, Vienna, Austria
IAEA Vienna
Copyright American Society of Nuclear Cardiology

## Slide 2
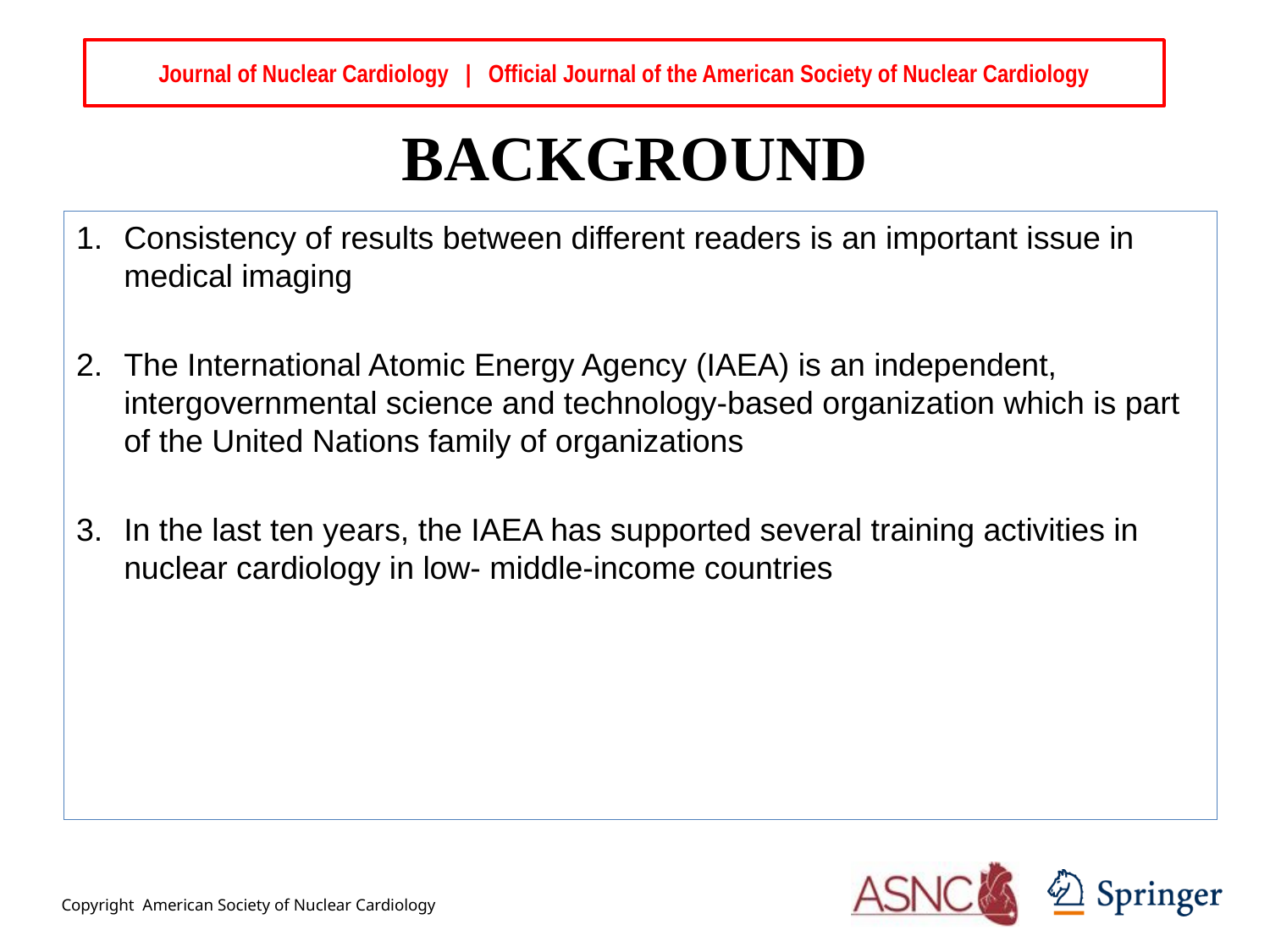

Journal of Nuclear Cardiology | Official Journal of the American Society of Nuclear Cardiology
# BACKGROUND
Consistency of results between different readers is an important issue in medical imaging
The International Atomic Energy Agency (IAEA) is an independent, intergovernmental science and technology-based organization which is part of the United Nations family of organizations
In the last ten years, the IAEA has supported several training activities in nuclear cardiology in low- middle-income countries
Copyright American Society of Nuclear Cardiology

## Slide 3
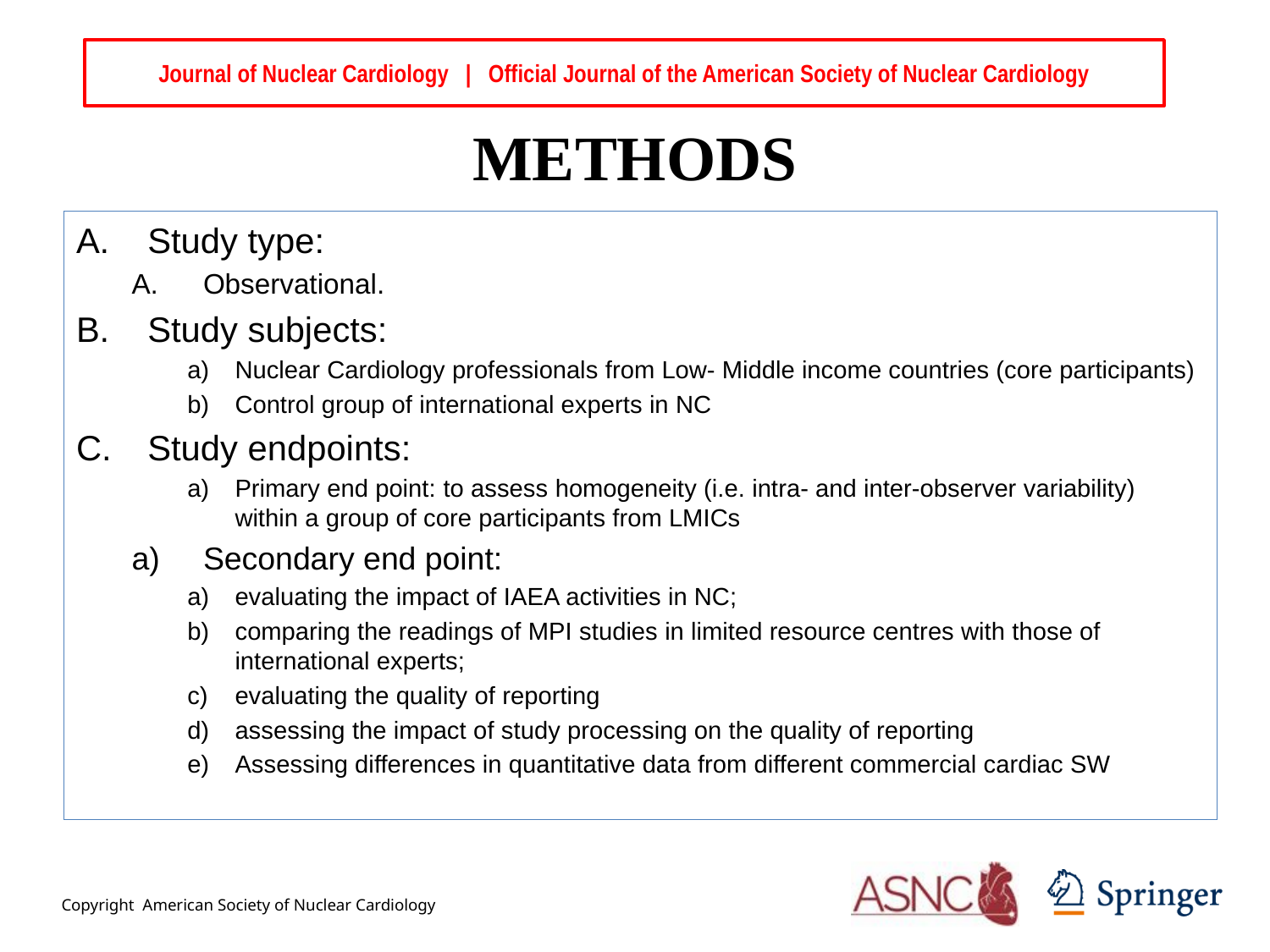

Journal of Nuclear Cardiology | Official Journal of the American Society of Nuclear Cardiology
# METHODS
Study type:
Observational.
Study subjects:
Nuclear Cardiology professionals from Low- Middle income countries (core participants)
Control group of international experts in NC
Study endpoints:
Primary end point: to assess homogeneity (i.e. intra- and inter-observer variability) within a group of core participants from LMICs
Secondary end point:
evaluating the impact of IAEA activities in NC;
comparing the readings of MPI studies in limited resource centres with those of international experts;
evaluating the quality of reporting
assessing the impact of study processing on the quality of reporting
Assessing differences in quantitative data from different commercial cardiac SW
Copyright American Society of Nuclear Cardiology

## Slide 4
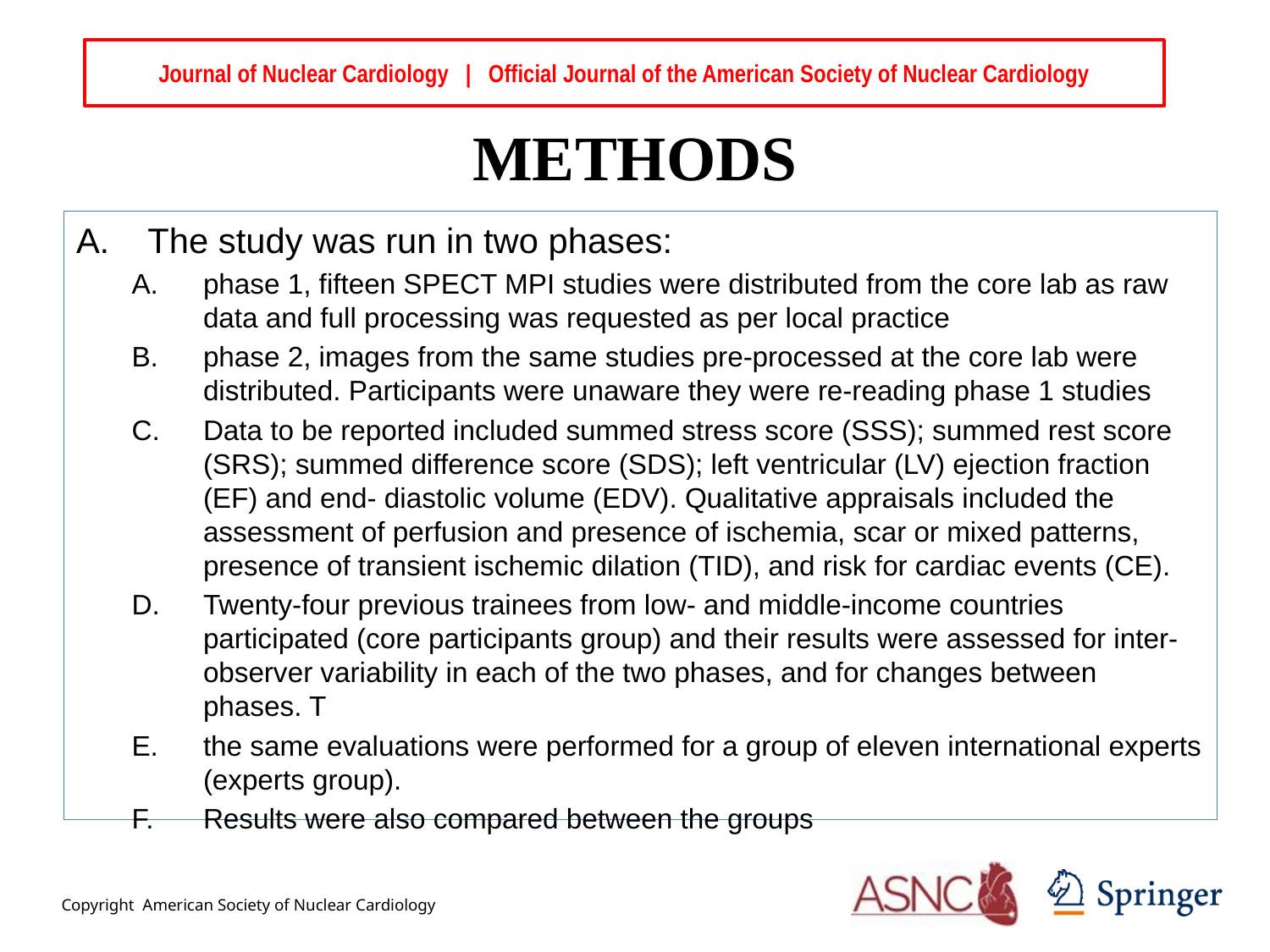

Journal of Nuclear Cardiology | Official Journal of the American Society of Nuclear Cardiology
# METHODS
The study was run in two phases:
phase 1, fifteen SPECT MPI studies were distributed from the core lab as raw data and full processing was requested as per local practice
phase 2, images from the same studies pre-processed at the core lab were distributed. Participants were unaware they were re-reading phase 1 studies
Data to be reported included summed stress score (SSS); summed rest score (SRS); summed difference score (SDS); left ventricular (LV) ejection fraction (EF) and end- diastolic volume (EDV). Qualitative appraisals included the assessment of perfusion and presence of ischemia, scar or mixed patterns, presence of transient ischemic dilation (TID), and risk for cardiac events (CE).
Twenty-four previous trainees from low- and middle-income countries participated (core participants group) and their results were assessed for inter-observer variability in each of the two phases, and for changes between phases. T
the same evaluations were performed for a group of eleven international experts (experts group).
Results were also compared between the groups
Copyright American Society of Nuclear Cardiology

## Slide 5
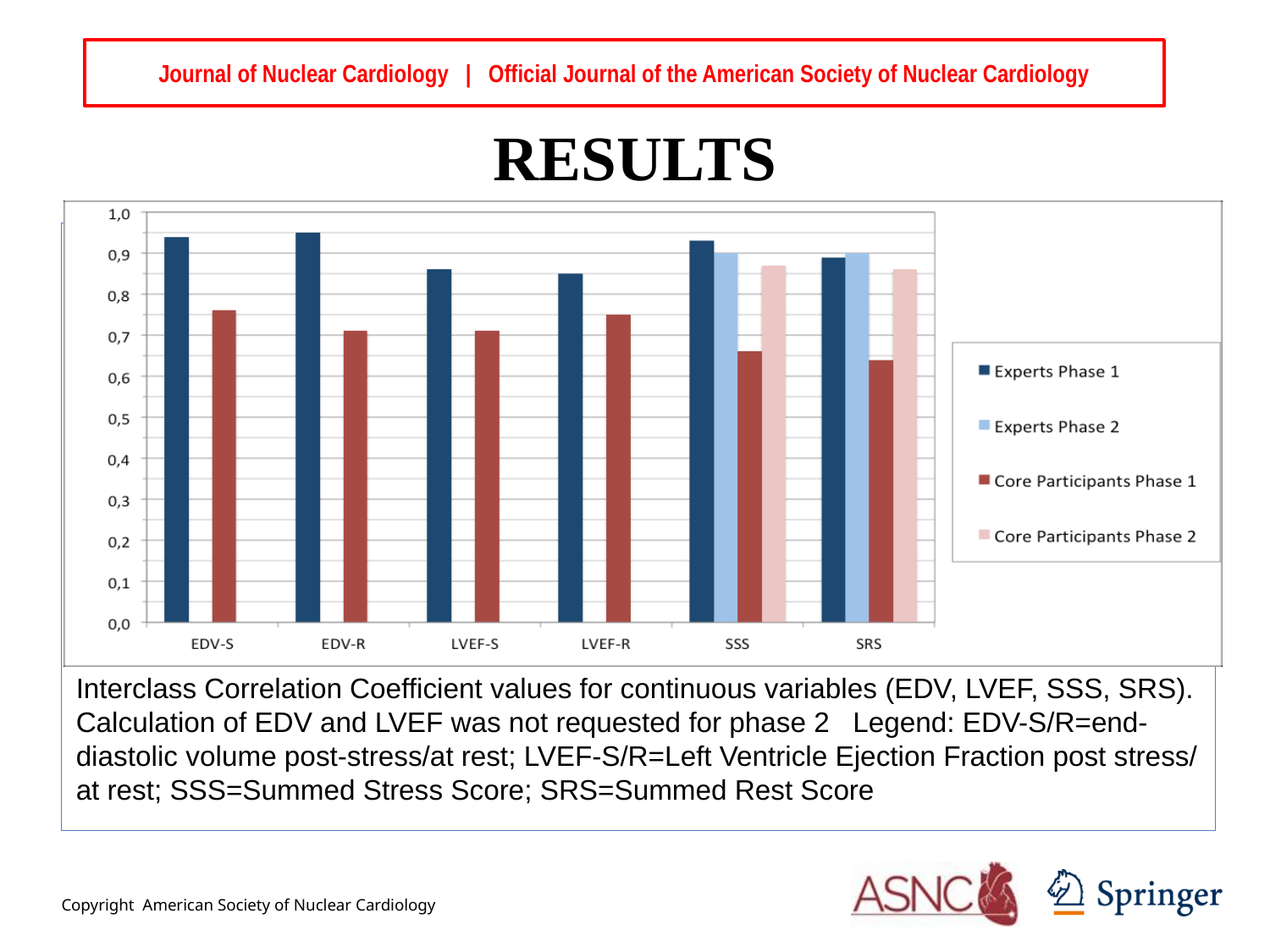

Journal of Nuclear Cardiology | Official Journal of the American Society of Nuclear Cardiology
# RESULTS
Interclass Correlation Coefficient values for continuous variables (EDV, LVEF, SSS, SRS). Calculation of EDV and LVEF was not requested for phase 2 Legend: EDV-S/R=end-diastolic volume post-stress/at rest; LVEF-S/R=Left Ventricle Ejection Fraction post stress/ at rest; SSS=Summed Stress Score; SRS=Summed Rest Score
Copyright American Society of Nuclear Cardiology

## Slide 6
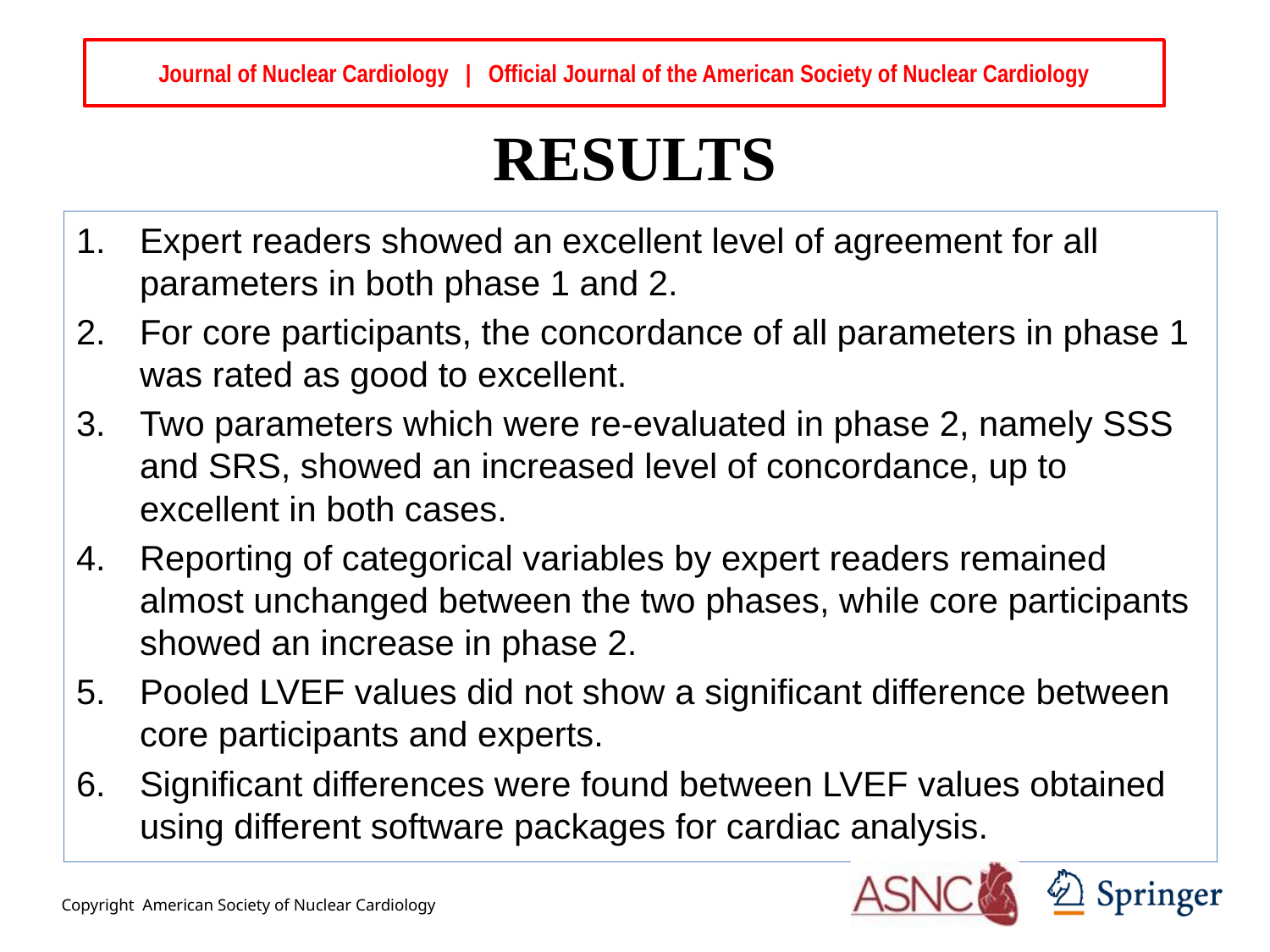

Journal of Nuclear Cardiology | Official Journal of the American Society of Nuclear Cardiology
# RESULTS
Expert readers showed an excellent level of agreement for all parameters in both phase 1 and 2.
For core participants, the concordance of all parameters in phase 1 was rated as good to excellent.
Two parameters which were re-evaluated in phase 2, namely SSS and SRS, showed an increased level of concordance, up to excellent in both cases.
Reporting of categorical variables by expert readers remained almost unchanged between the two phases, while core participants showed an increase in phase 2.
Pooled LVEF values did not show a significant difference between core participants and experts.
Significant differences were found between LVEF values obtained using different software packages for cardiac analysis.
Copyright American Society of Nuclear Cardiology

## Slide 7
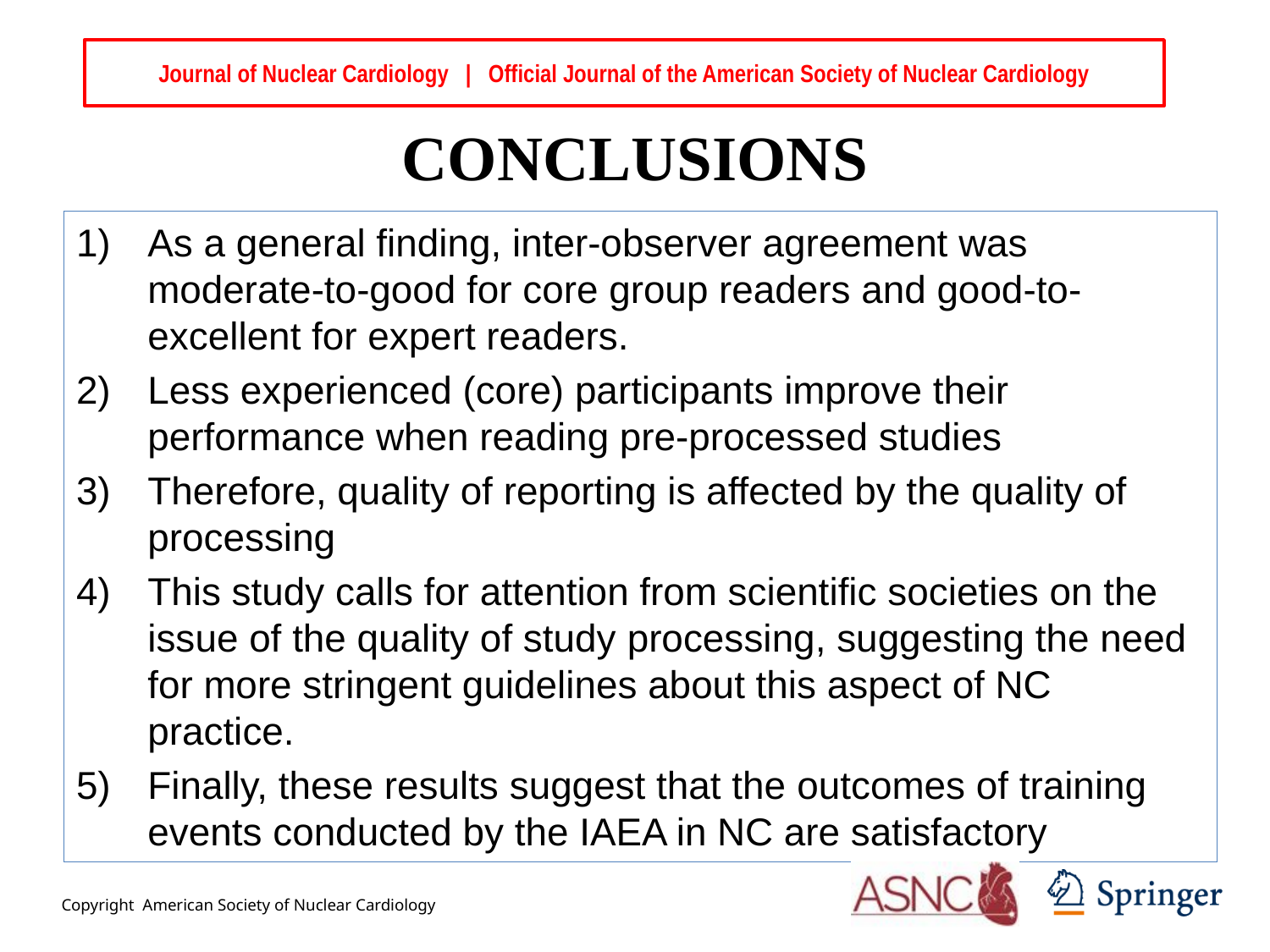

Journal of Nuclear Cardiology | Official Journal of the American Society of Nuclear Cardiology
# CONCLUSIONS
As a general finding, inter-observer agreement was moderate-to-good for core group readers and good-to-excellent for expert readers.
Less experienced (core) participants improve their performance when reading pre-processed studies
Therefore, quality of reporting is affected by the quality of processing
This study calls for attention from scientific societies on the issue of the quality of study processing, suggesting the need for more stringent guidelines about this aspect of NC practice.
Finally, these results suggest that the outcomes of training events conducted by the IAEA in NC are satisfactory
Copyright American Society of Nuclear Cardiology
